# Biogenic Synthesis, Characterization and Antibacterial Potential Evaluation of Copper Oxide Nanoparticles Against *Escherichia coli*

**DOI:** 10.1186/s11671-021-03605-z

**Published:** 2021-09-20

**Authors:** Mohsin Ali, Muhammad Ijaz, Muhammad Ikram, Anwar Ul-Hamid, Muhammad Avais, Aftab Ahmad Anjum

**Affiliations:** 1 Department of Veterinary Medicine, University of Veterinary and Animal Sciences, Lahore, Punjab 54000 Pakistan; 2 Solar Cell Applications Research Lab, Department of Physics, Government College University Lahore, Lahore, Punjab 54000 Pakistan; 3 Core Research Facilities, King Fahd University of Petroleum and Minerals, Dhahran, 31261 Saudi Arabia

**Keywords:** Bactericidal, Nanoparticles, Doping, *E. coli*, Mastitis, Biogenic synthesis, Ginger, Garlic

## Abstract

The development of resistance against antibiotics used to treat bacterial infections along with the prevalence of medication residues presents significant public health problems globally. Antibiotic-resistant germs result in infections that are difficult or impossible to treat. Decreasing antibiotic effectiveness calls for rapid development of alternative antimicrobials. In this respect, nanoparticles (NPs) of copper oxide (CuO) manifest a latent and flexible inorganic nanostructure with noteworthy antimicrobial impact. Green synthesis of CuO NPs was performed in the current study, which was then doped with varying amounts of ginger (*Zingiber officinale*, ZO) and garlic (*Allium sativum*, AS) extracts. In low and high doses, the synthesized compound was used to measure the antimicrobial effectiveness against pathogenic *Escherichia coli*. The present research successfully demonstrated a renewable, eco-friendly synthesis technique with natural materials that is equally applicable to other green metal oxide NPs.

**Supplementary Information:**

The online version contains supplementary material available at 10.1186/s11671-021-03605-z.

## Introduction

Indigenous herbal and spicy plants include valuable antioxidant and antibacterial properties that are employed in human and veterinary treatment [1]. Medicinal plants containing antimicrobial properties include garlic, ginger, neem, turmeric, tulsi, etc. and among these, garlic and ginger are famous for their medicinal use [2]. *Zingiber officinale* commonly known as ginger is an indigenous plant root belonging to Zingiberaceae family. It contains gingerol, shogaol, curcumin and paradol as important phytochemicals [3]. Antimicrobial activity against a wide range of microbes has been manifested by aqueous extracts of ginger, owing to its phenolic contents of therapeutic nature [4]. *Allium sativum* commonly known as garlic contains phenolic compounds exhibiting a broad-spectrum antibacterial activity even against MDR bacteria [5]. *Allium sativum* has exhibited broad-spectrum antibacterial activity against a number of Gram-positive and various Gram-negative bacteria [6].

Exploiting the knowledge of nanotechnology at molecular and atomic levels serves as a basis to apply an integrative approach to develop novel compounds with unique features for use in broad-spectrum applications [7]. Medicinal, agricultural, food preservative and cosmetic applications of nanoparticles due to their unmatched inherent properties have led to an increased exploration by researchers [8, 9]. Various biological applications of copper oxide nanoparticles have been successfully demonstrated including potential antimicrobials, effective therapeutic compounds, drug delivery carriers, photocatalysts, gas sensing, photovoltaic stability, quantum confinement effect and biological probes [10–14]. Reactive oxygen species (ROS) generation is triggered by nanoparticles due to their semiconductor nature, bringing oxidative and degenerative transformations at the cellular level resulting in destruction of bacterial cell walls and release of cellular contents [15]. Many methods have been practiced for the synthesis of nanoparticles, i.e., chemical, physical and biological synthesis [16]. Reduction of metallic compounds results in the production of nanoparticles using any biochemical or microorganism, plants or their extracts [17].

*Escherichia coli* (*E. coli*), a natural inhabitant of intestine and part of intestinal flora, has a distinctive position in microbiological world due to its potential virulent properties [18]. The existence of virulence depends on the number of genes in *E. coli* isolates and in some cases horizontal transfer of resistance genes has also been revealed [19, 20] which may create a health concern in human beings, animals [21] and challenge for food safety and security [22]. *E. coli* is causative agent of mastitis in dairy cows and buffaloes and was found responsible for a major decline in milk yield and consequent economic losses [23, 24], which developed resistance genes, i.e., extended-spectrum β-lactamases (ESBLs) or over-expressed cephalosporinases (AmpCs) [25]. Treatment failure associated with *E. coli* infections is considered as a latent threat leading to multidrug resistance both in human and veterinary medicines [26].

Nanoparticles with their characteristic antimicrobial features have the potential to kill around 600 cells in contrast to antibiotic’s ability to treat only a few diseases of infectious origin [27]. The current study aims to explore, evaluate and compare the possible antimicrobial potential of green and chemically synthesized CuO nanoparticles and extracts of common herb roots of *Allium sativum* (AS) and *Zingiber officinale* (ZO), against pathogenic *E. coli* as alternatives to antibiotics to overcome the emerging resistance challenges.

## Methods

The current study was aimed at investigating the bactericidal action of phytochemically reduced CuO NPs against herb roots of *Allium sativum* (AS) and *Zingiber officinale* (ZO), an isolate of bovine mastitis.

### Materials

Chemically manufactured nanoparticles of CuO were procured from Sigma-Aldrich, whereas ZO and AS roots were acquired from local fruits and vegetable market of Lahore, Pakistan. Roots of ZO and AS were dried off under shade to attain even weight. Growth media for *E. coli* and analytical grade chemicals were utilized without modification.

### Aqueous Extraction of ZO and AS Roots

**Fig. 1:**
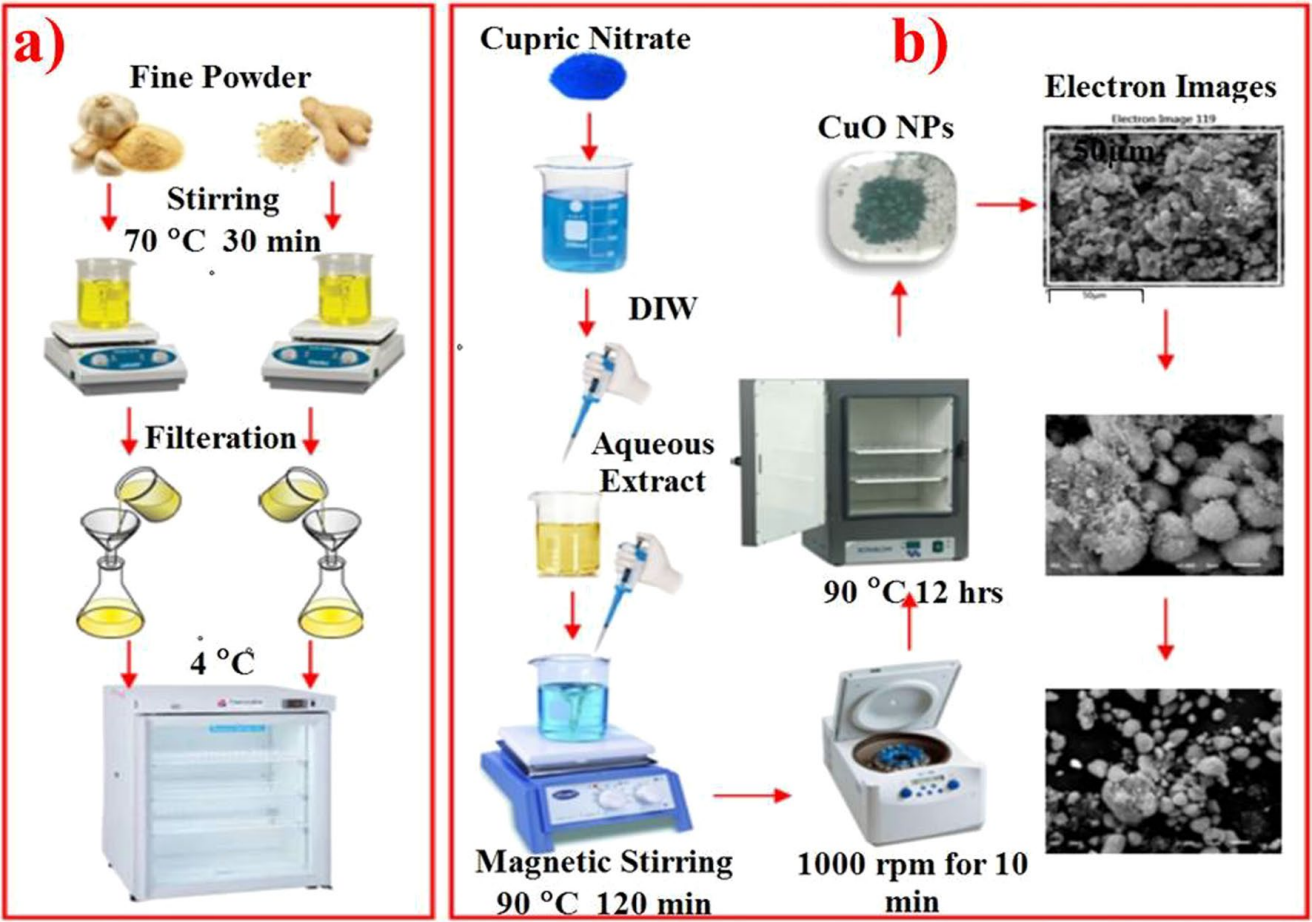
Overview of **a** aqueous extraction of *Zingiber officinale* and *Allium sativum* roots, **b** green synthesis of CuO NPs

Dried roots of ZO and AS were ultrafine ground to fine powders that were then stored in airtight jars. Robust stirring of 30 min at 70 °C for mixing of fine powdered roots with distilled water-DIW was undertaken at 1:10 ratio. Filtration of the prepared solutions was performed using Whatman filter paper No. 1 after cooling the solutions and the storage of the filtrate was carried out at 4 °C for the next experiment [28] as exhibited in Fig. 1a.

### Green Synthesis of CuO

Cupric nitrate tetrahydrate (0.1 M) was blended with different concentrations, i.e., 3 mL, 6 mL and 12 mL of ZO and AS extracts under continuous stirring. NaOH (2 M) was used to maintain pH 12 of the stirred solution for 2 h at 90 °C for the formation of precipitates. Centrifugation of the precipitates was carried out at 10,000 rpm for 20 min, followed by DI water washing and overnight drying in a hot air oven at 90 °C [29] as portrayed in Fig. 1b.

### Characterization

UV–visible GENESYS-10S spectrophotometer was used to check the absorption spectra of CuO nanoparticles and aqueous extracts of ZO and AS between wavelengths range of 200 to 500 nm [30]. CuO NPs structural and phase analysis was performed using X-ray diffraction (XRD) BRUKER D2 Phaser with 2*θ* = (10°–80°) equipped with Cu Kα radiation of *λ* = 1.540 Å [31]. Fourier transform infrared spectroscopy (ATR-FTIR) was performed for the analysis of functional groups in CuO NPs and aqueous extracts of ZO and AS [31]. Scanning electron microscopy (JSM-6610LV SEM) coupled with EDS detector was undertaken for the observation of elemental and morphological constitution of CuO NPs. High-resolution TEM images and SAED patterns were taken using JEOL JEM-2100F microscope [32].

### *E. coli* Isolation and Identification

#### Collection of Samples

Cows and buffaloes suffering from clinical mastitis were traced and identified from various livestock farms for the collection of milk samples.

#### *E. coli* Isolation

MacConkey agar was used for streaking and culturing of milk in triplicate for isolation of purified colonies of *E. coli* [33]. Disk diffusions of distinctive colonies of *E. coli* were evaluated to check susceptibility against specific antibiotics observing the guidelines of the National Committee for Clinical Laboratory Standards (NCCLS) to isolate *E. coli*.

#### Identification of *E. coli*

Identification and confirmation of *E. coli* colonies were carried out based on Gram’s staining; distinguishing morphological characters and biochemical tests, i.e., methyl red and catalase tests with perspective to the Bergey's Manual of Systematic Bacteriology. Culturing of isolates on eosin methylene blue (EMB) agar was performed for *E. coli* distinctness and ratification from related Gram-negative mastitogens.

### Evaluation of CuO NPs In Vitro Antibacterial Potential Against *E. coli*

A number of experiments were performed to evaluate *in vitro* antibacterial potential of extract doped CuO NPs against pathogenic *E. coli*. *In vitro* trials were conducted using 10 representative pathogenic *E. coli* isolates for the evaluation of antibacterial potential of CuO NPs. Disk diffusion method assessment was used to assess *in vitro* antimicrobial potential. Petri dishes were swabbed with activated growth of *E. coli* 1.5 × *108* CFU/mL (0.5 *Mcfarland* standard) on MacConkey agar [34]. A sterile cork borer was used to prepare 6-mm-diameter wells in the petri dishes. Aqueous extracts of ZO and AS*,* along with green doped extracts and chemically synthesized copper oxide nanoparticles in different concentrations, were applied into wells. Antimicrobial potential of aqueous extracts of ZO and AS along with green doped extracts and chemically synthesized copper oxide nanoparticles was evaluated by aerobically incubating the petri dishes at 37 °C overnight by measuring the zones of inhibition (mm) using Vernier caliper. Statistical analysis of zones of inhibition (mm) was performed using one-way ANOVA and visualizing (*p* < 0.05).

## Results and Discussion

**Fig. 2:**
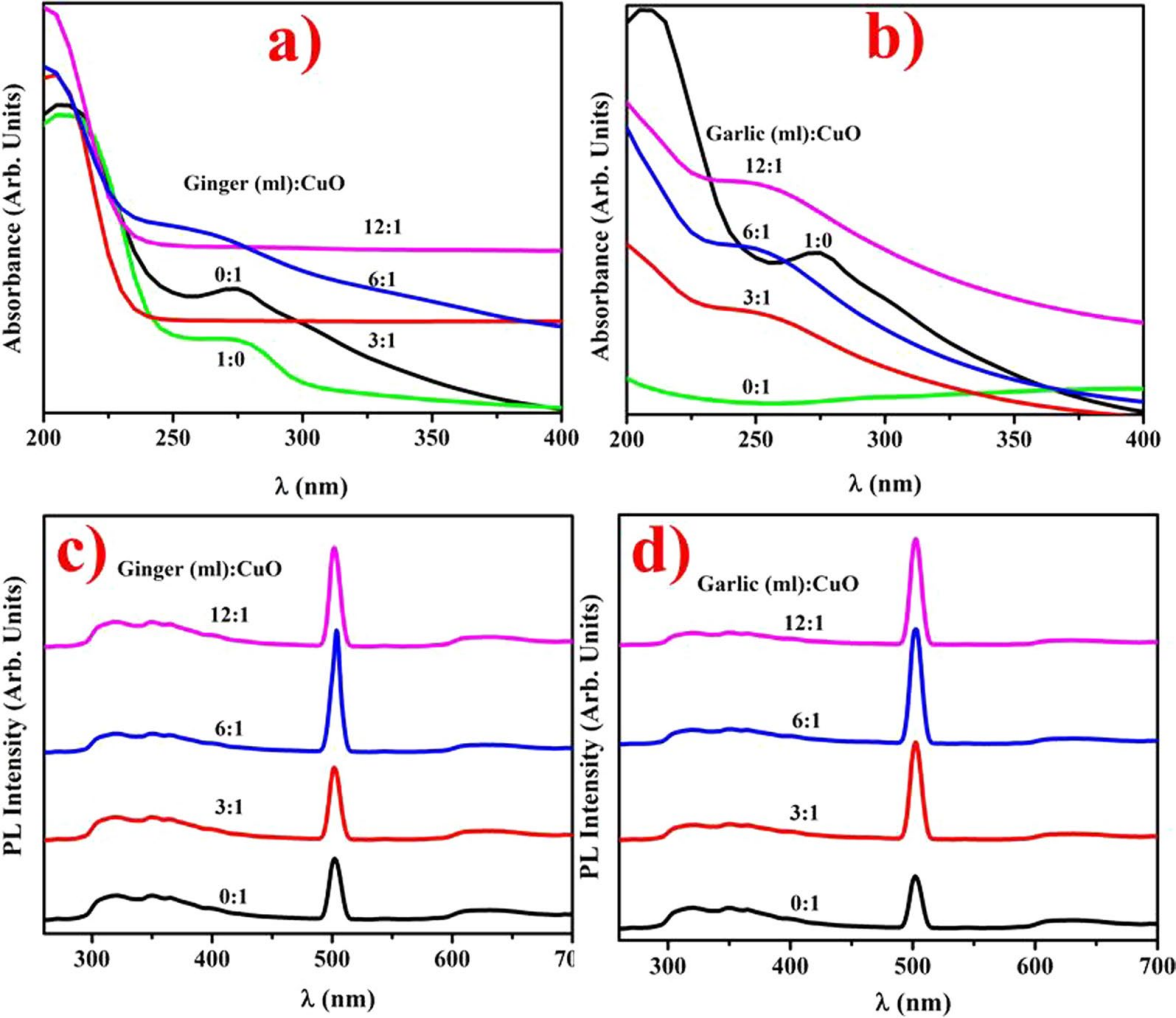
Absorption spectra of CuO NPs doped with **a** ZO **b** AS extract and PL spectra CuO NPs with **c** ZO and **d** AS extracts, respectively

UV–Vis spectroscopy of doped CuO NPs and aqueous extracts of ZO and AS was accomplished to investigate the optical behavior as shown in Fig. 2a, b. For subsequent formation of CuO NPs, gradual color shift from wine to coal black was noticed while optimization of synthesized NPs by means of vaulted, acoustic extracts was undertaken. Aqueous extracts ZO and AS absorption peaks at 275 and 280 nm were observed. Results depicted *λ*_max_ for ZO- and AS-doped CuO at 250 nm depicting ratio 6 mL:1 with characteristic redshifts and blueshifts, respectively [35]. Wide peaks specified particle clusters and transition of electrons to conduction bands from valence phase with concentrated extracts in CuO as divulged by strong absorption bands [36]. In green synthesized CuO NPs, reduced absorption was seen by increasing or reducing extract volume in addition to the optimized value (6 mL:1).

Additionally, it was remarkable to observe that semiconducting structures confirmed their physical features to the existing nanometric quantum-size effects. PL spectra of ZO- and AS-doped CuO NPs with exciting UV wavelength of 300 nm are presented in Fig. 2c, d. The three emission peaks at 418, 561 and 664 nm were represented on each pure and doped PL graph of CuO (UV region) [37]. A violet–blue light band found at 418 nm is a standard CuO emission peak at the near band edge [38, 39]. At 430 nm, the shoulder edge can be due to CuO vacancies, which is a p-type semiconductor. The yellowish green edge is responsible for the depth defects under low temperatures at 561 nm. The red emission peaks at 664 nm are responsible for different copper conditions or the presence of individual ionized oxygen vacancies [40, 41]. The diverse existence of visible emissions in the violet–blue, yellow–green and red spectrum indicates that the studied CuO particles have a high volume–surface ratio and a multitude of surface-to-volume conditions and defects (vacancies or interstitials) that produce trap-to-emission ranges [40, 42].

**Fig. 3:**
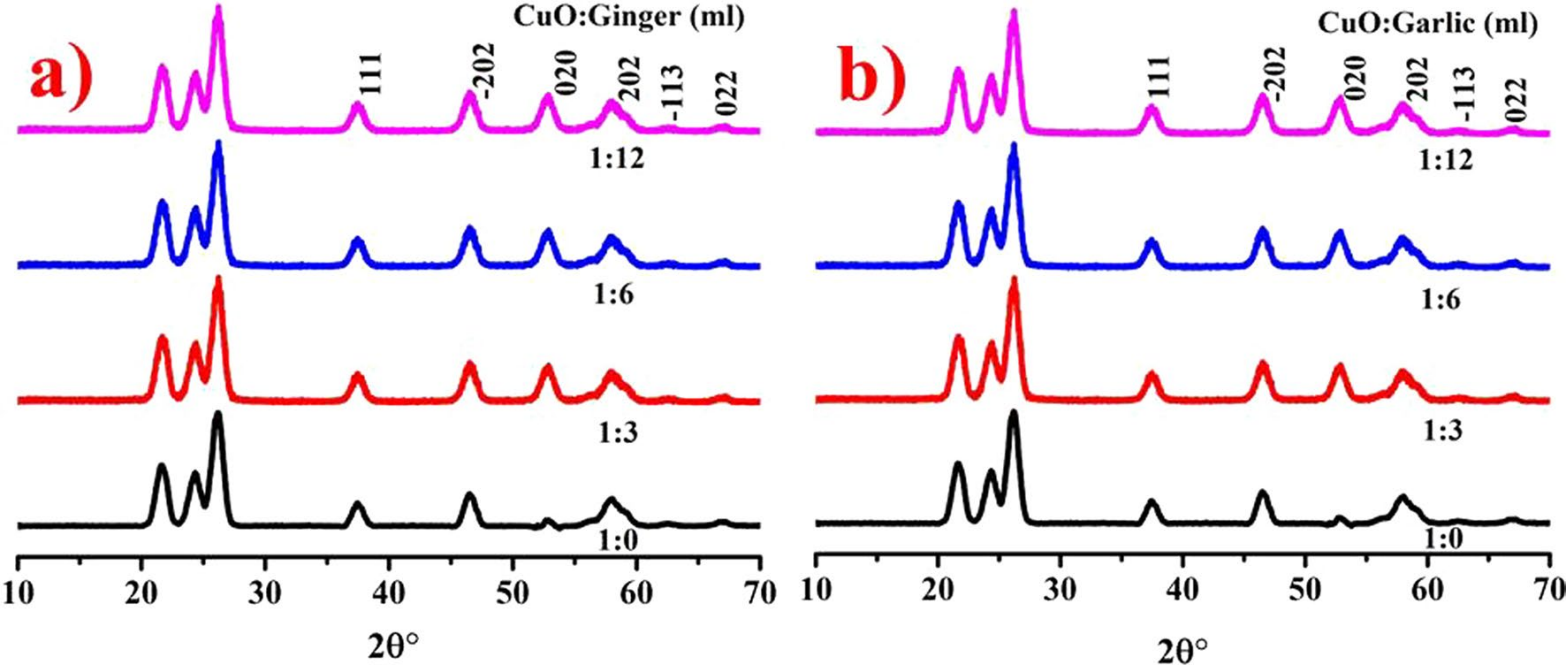
XRD patterns of CuO NPs without and with doping of **a** AS **b** ZO

XRD was performed to assess the crystalline structure, composition and scale of CuO NPs as presented in Fig. 3a, d doped with ZO and AS, respectively. The increase in crystallinity was demonstrated by peaks detected at 2*θ* = 38.7°, 48.6°, 53.5°, 58.3°, 61.7° and 66.2° with corresponding crystal planes (111), (−202), (020), (202), (−113) and (022), respectively. The detected peaks assured the presence of CuO monoclinic phase as synchronized with JCPDS card no: 00–002–1040 [43]. The characteristic crystallite size measured using *D* = 0.9*λ*/*β*cos*θ* was found to be 24.7 and 47.6 nm for ZO- and AS-doped CuO, respectively, and pristine sample crystallite size was 27.4 nm. Several natural products have been identified as capping and reducing agents for average crystallite size in AS and ZO extracts [44].

**Fig. 4:**
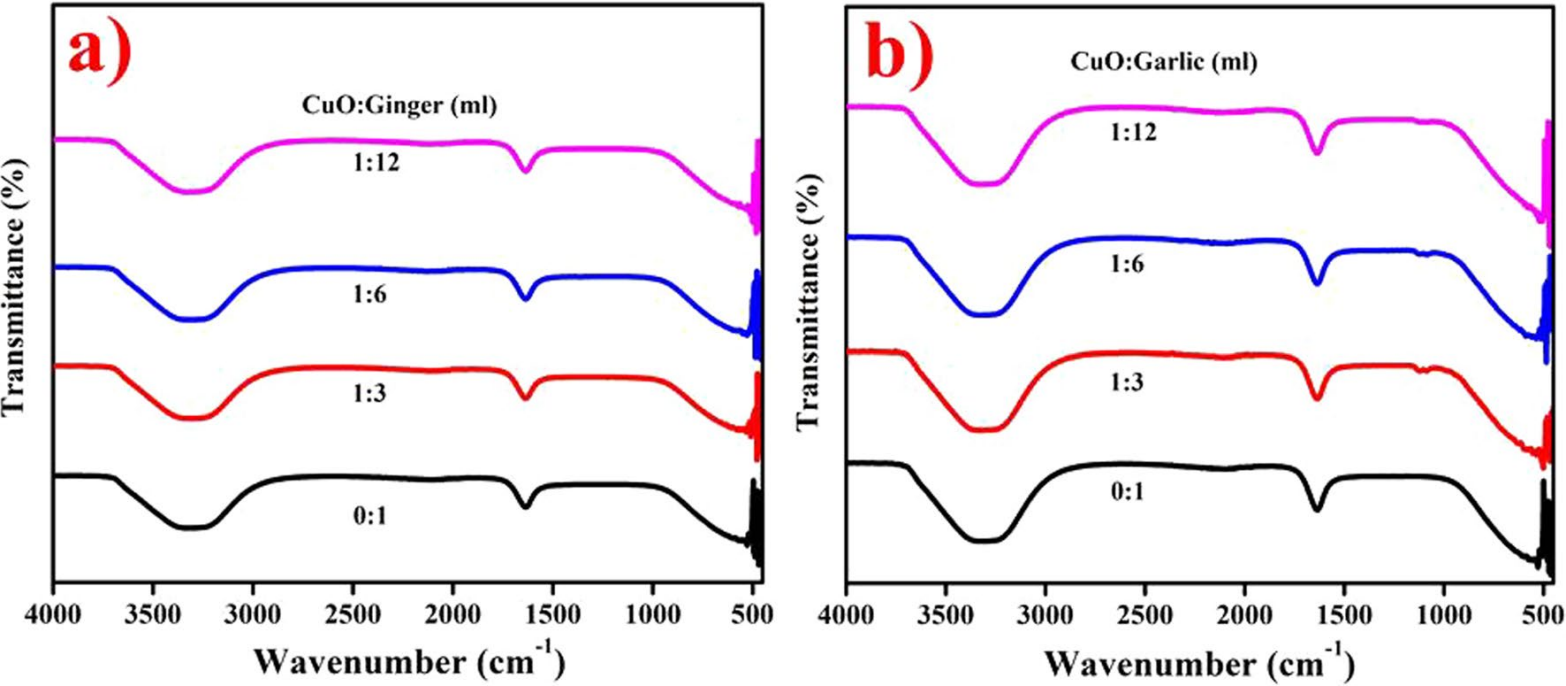
FTIR spectra of **a** AS **b** ZO-doped CuO NPs

Functional groups of AS and ZO extract doped CuO NPs were examined with FTIR as illustrated in Fig. 4a, b, correspondingly. The broad peak at 3314 cm^−1^ endorsed the presence of hydroxyl group and peak wideness represents direct C=O with (N–H) amines [45]. The intense peak at 1638 cm^−1^ corresponded to CH_2_–OCH_3_ group existing in 6-snogal and 6-gingerol of ZO as found substantial reduction of CuO. The typical single bonds of Cu–O seen at 478.8 cm^−1^ in the twisting fashion were due to strong modes of vibration [46]. All peaks suggest that alcohol, amine and ketone groups resulted in chelation and capping of flavonoids, plant chemical substances and proteins [47].

**Fig. 5:**
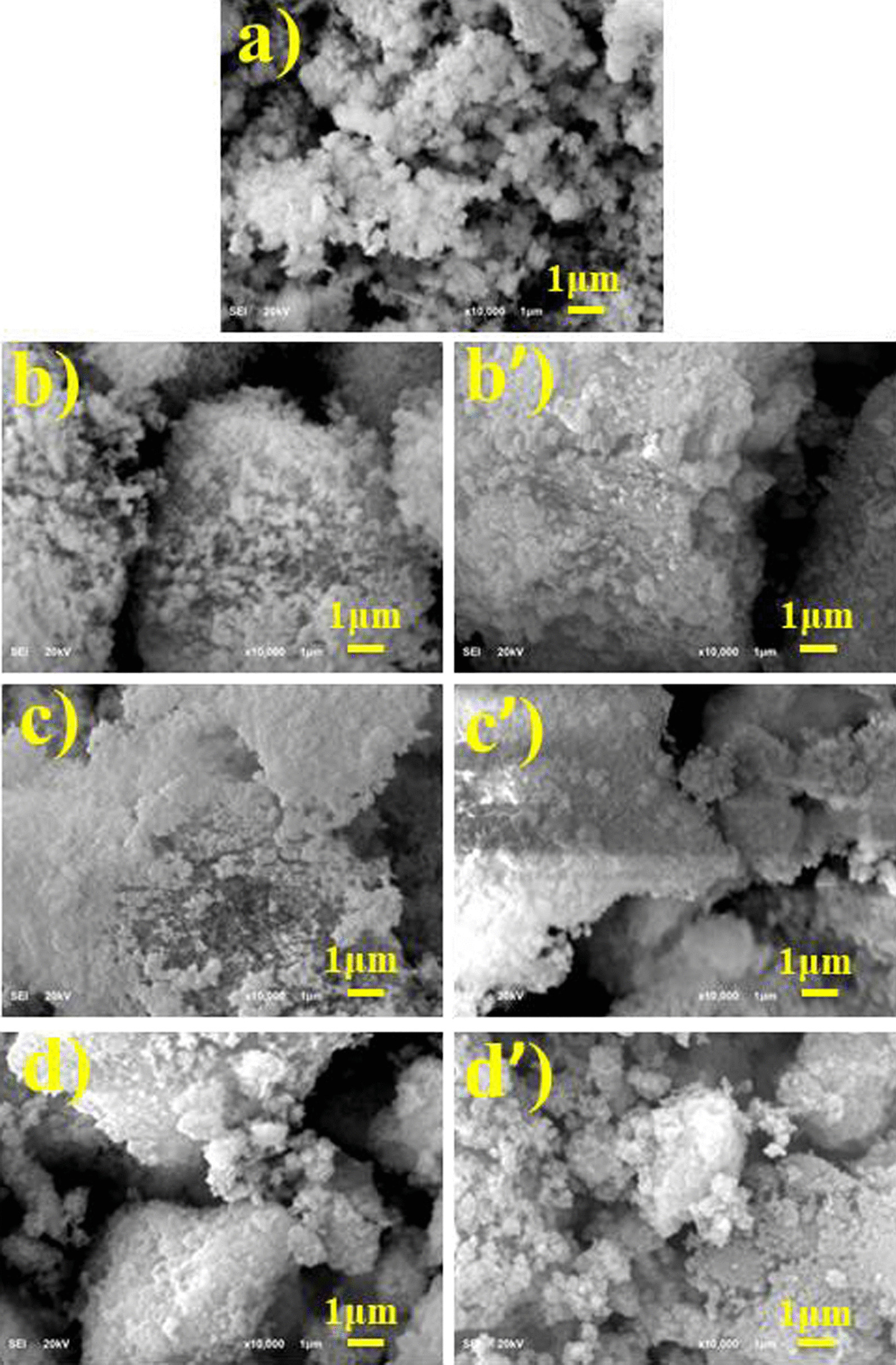
Images CuO NPs taken with FESEM **a** CuO, **b**–**d** ZO-doped CuO and **b′**–**d′** AS-doped CuO NPs, respectively

FE-SEM was used to study surface characteristics and scale of CuO NPs doped with ZO and AS extract as shown in Fig. 5a–d′. The FE-SEM images show that the CuO NPs are extremely agglomerated in spherical morphology. Magnetic interference and the conformity of polymers between particles may show agglomeration between particles [48]. ZO and AS doping with CuO was evident by the images showing cluster formation and particle size seems < 1 μm as illustrated in Fig. 5b–d′.

**Fig. 6:**
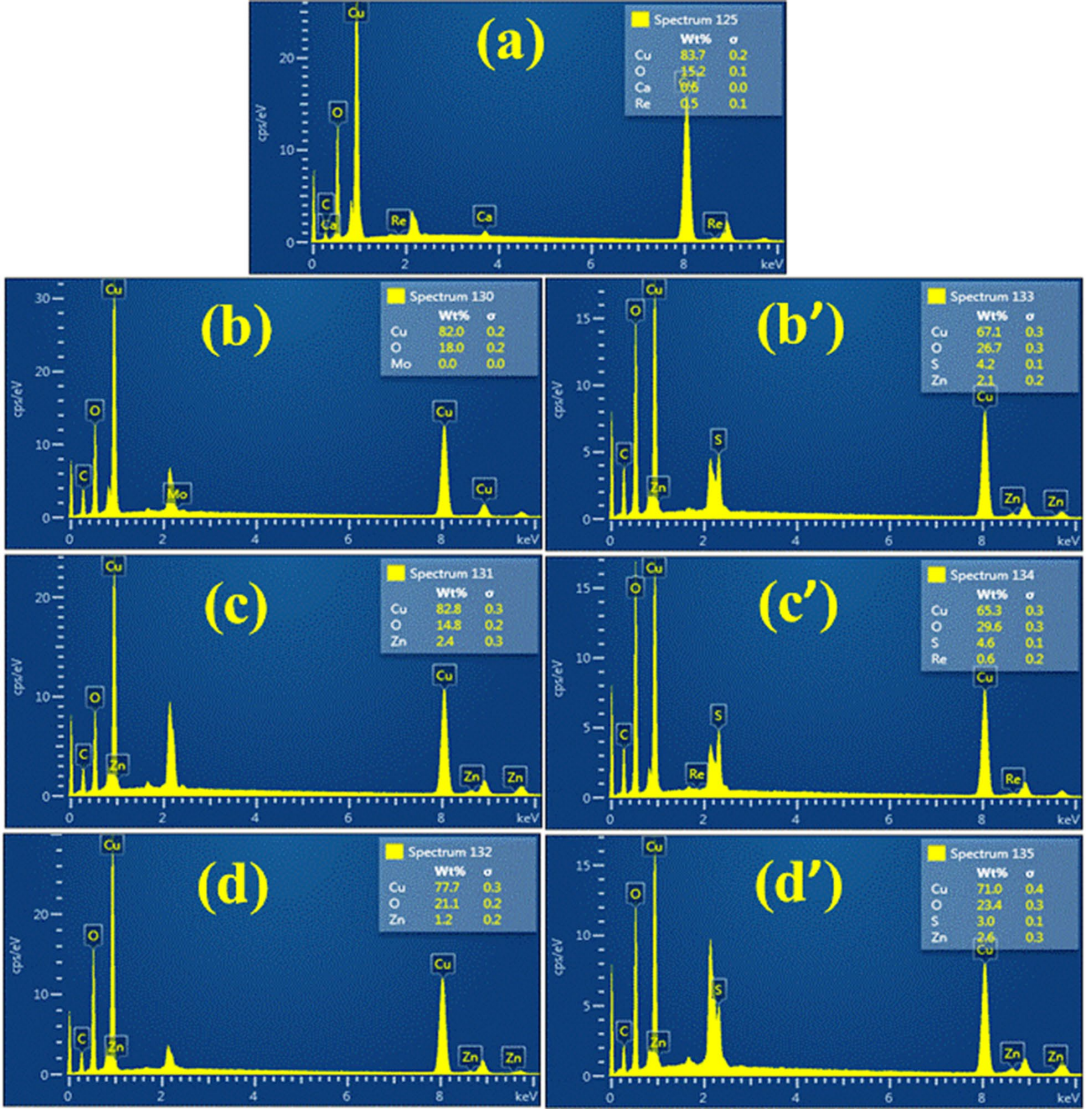
EDS spectra of CuO NPs **a** pure CuO **b**–**d** ZO-doped CuO and **b′**–**d′** AS-doped CuO NPs, respectively

Energy-dispersive X-ray spectroscopy (EDS) illustrated the chemical composition with elemental investigation of pristine sample and doped CuO NPs with ZO and AS root extracts by inveterate CuO phases as shown in Fig. 6a–d′. Three peaks corresponding to the high purity of Cu confirmed by EDS of the samples tested in comparison with precursor oxygen between 1 and 10 keV. CuO NPs surface plasmon resonance (SPR) resulted in absorption peaks [49]. 83.7%, 15.2% and 0.6% were observed for atomic weight through spectra for Cu, O and Ca, respectively, for control sample whereas 82.8%, 14.8% and 2.4% for the ZO-doped and optimized sample (6 mL:1) observed through spectra for Cu, O and Zn, respectively. Similarly, 65.3, 29.6 and 4.6 for Cu, O and S with AS doping were found, respectively. The supplementary atomic compounds appearing in EDS responded to SEM sample holder used during analysis [50].

**Fig. 7:**
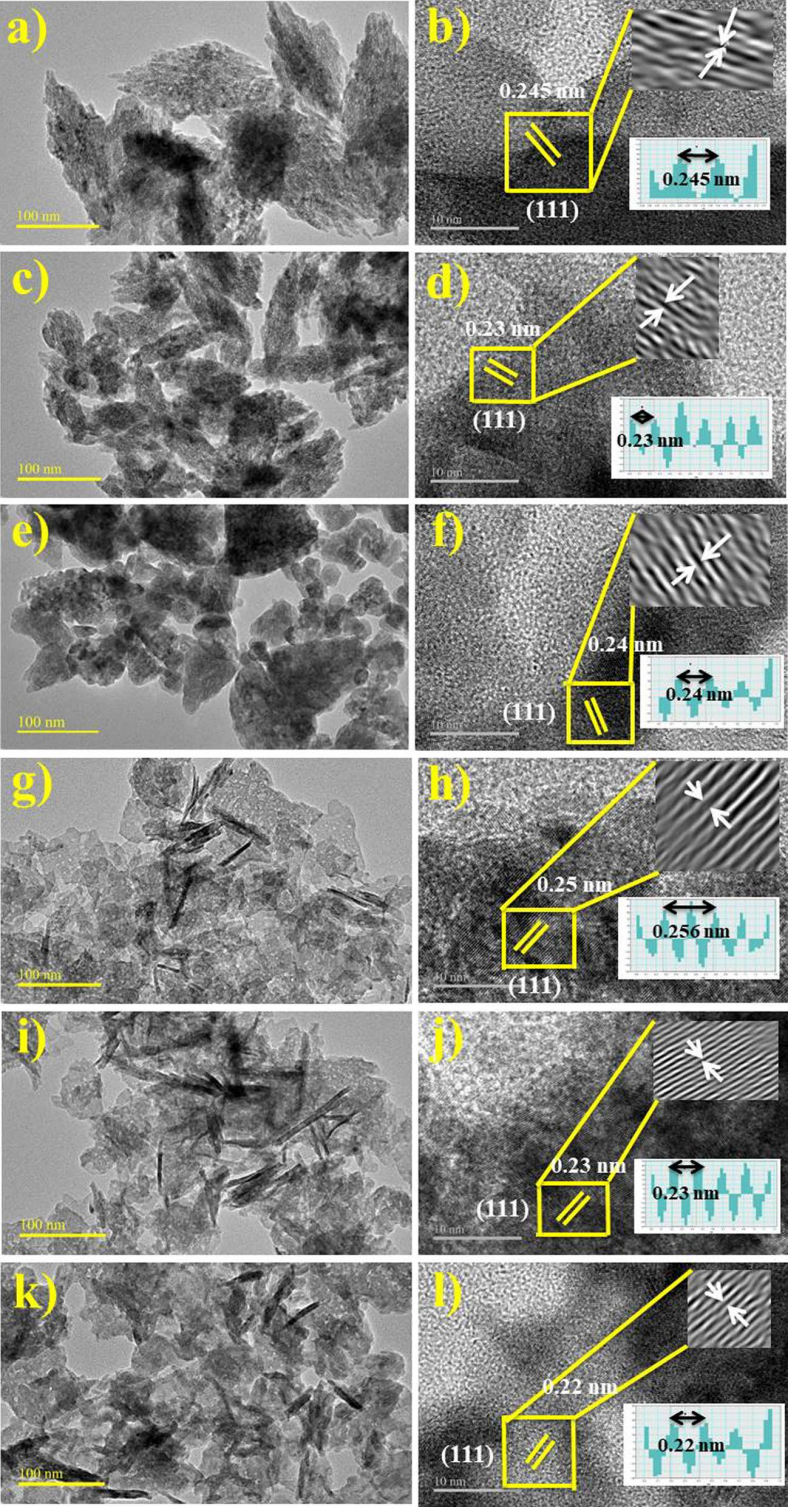
**a**, **b** HR-TEM, **a**–**f** lattice fringes of 3 mL, 6 mL and 12 mL ZO-doped CuO **g**–**l** lattice fringes of 3 mL, 6 mL and 12 mL AS-doped CuO NPs, respectively

The exclusive and characteristic structure of CuO NPs was further assessed using HR-TEM at 50 nm as displayed in Fig. 7a–l. HR-TEM pictures revealed adorned nanoparticles similar to FE-SEM images, along with higher agglomeration while size measuring less than 50 nm. The presence of phytochemicals in ZO- and AS-doped CuO NPs were also confirmed with HR-TEM images [51]. Neither any imperfection nor any deformity was observed in the integral lattice structure of ZO- and AS-doped CuO NPs [52]. Filtered micrographs were presented by HR-TEM results along with fast Fourier transform [FFT] of specified area depicted by yellow square in Fig. 7b, d, f, h, j, l presenting high resolution structural and atomic characteristics. The HR-TEM mean particulate dimensions are precisely matched to the crystallite sizes observed during XRD and SEM analysis [53].

**Fig. 8:**
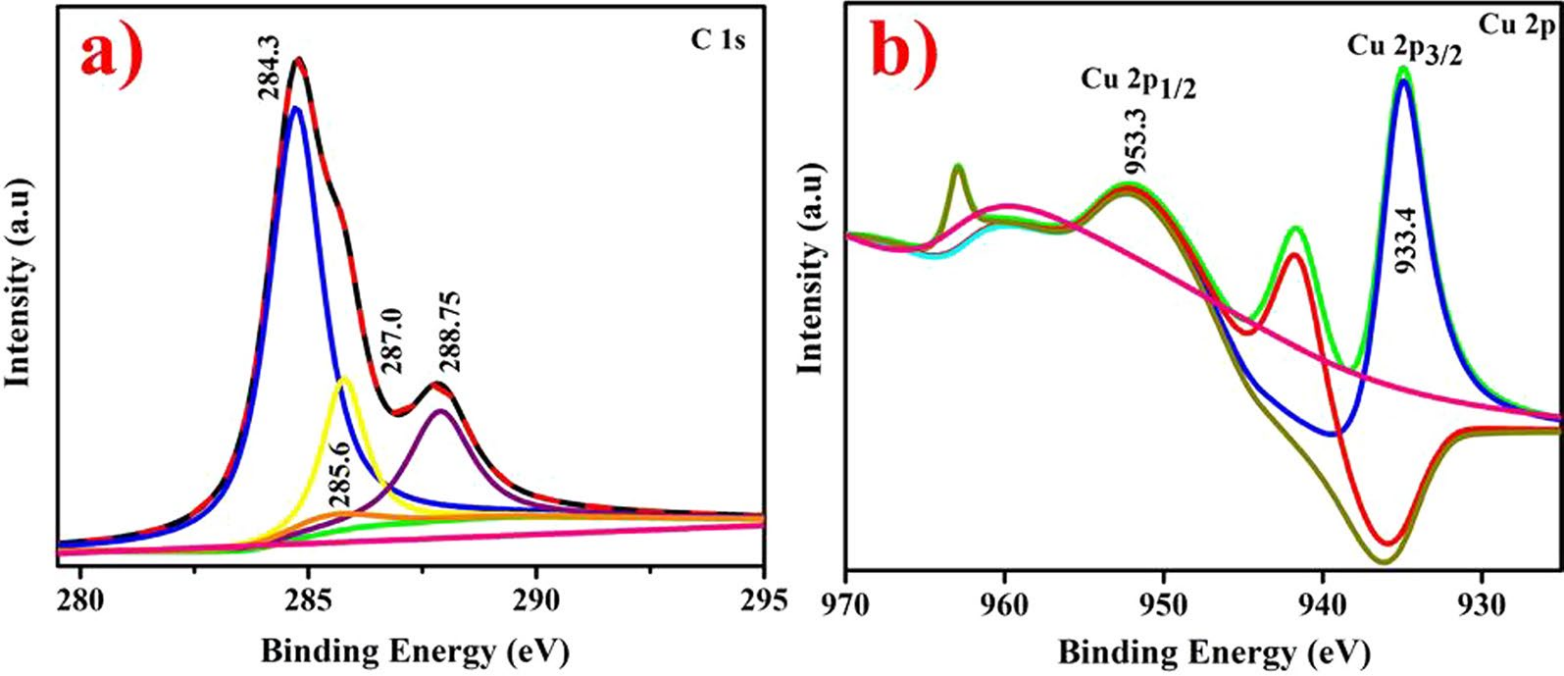
**a**, **b** XPS analysis of doped CuO NPs **a** C1s **b** Cu 2p

The XPS analysis of doped CuO with Gi and Ga CAE depicting C 1*s* and Cu 2*p* spectrum is shown in Fig. 8a, b. The C1*s* range indicated presence of four distinct peaks Fig. 8a with distinctive functional groups as C(H,C) (284.39 eV), C(N) (285.6 eV), C(O,=N) (287.0 eV) and C–O–C (288.75 eV) [54–56]. Mainly, Fig. 8b depicted Cu 2*p* pattern of doped CuO with peaks at 933.3 and 953.3 eV binding energies corresponding to Cu 2*p*_3/2_ and Cu 2*p*_1/2_ spin orbit indicates divalent oxidation state of prepared sample. The relevant peaks at 942.2 and 962 eV relate to satellite peaks of Cu 2*p*_3/2_ and Cu 2*p*_1/2_ which seemed mainly due to partially filled 3*d*^9^ orbital in divalent oxidation state [57].

**Fig. 9:**
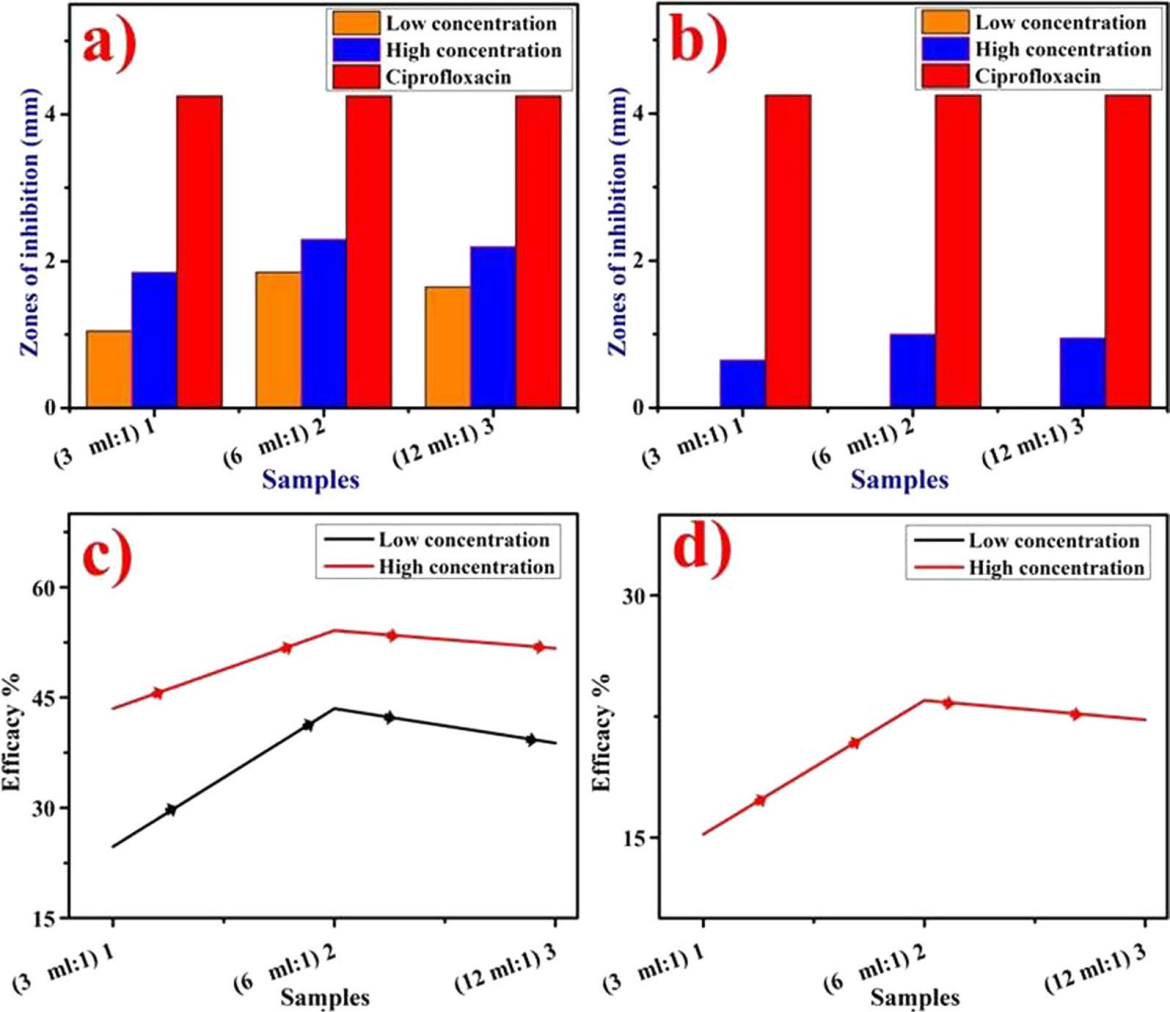
**a**–**d** In vitro antibacterial activity of CuO NPs **a** doped with ZO at ↓ and ↑ dose, **b** doped with AS at ↓ and ↑ doses, respectively, **c** efficacy %age doped with ZO and **d** efficacy %age doped with AS

Well diffusion method was applied for the evaluation of bactericidal potential of ZO and AS aqueous extracts and synthesized CuO NPs by measuring inhibition areas after incubating the petri plates for 24 h as shown in Fig. 9a–d, Additional file 1: Fig. S1 and tabulated in Table 1. The findings revealed that the NPs concentration and inhibiting zones responded synergistically. Significant inhibition zones found for sample 1 (3 mL:1), sample 2 (6 mL:1) and sample 3 (12 mL:1) were (1.05–1.85 mm) and (1.85–2.30 mm) using decreased (↓) and increased (↑) concentrations, respectively, for ZO-doped CuO nanoparticles (*p* < 0.05), Fig. 9a. Similarly, AS-doped NPs exhibited (0.65–1.00 mm) inhibition zones at maximum concentration only, Fig. 9b. AS-doped NPs demonstrated zero efficacy against pathogenic *E. coli* at minimum concentrations. ZO extract depicted effect at decreased (↓) concentration in comparison to increased (↑) concentration showing 1.55 mm zone, similarly, no antibacterial effect of AS extracts was found at both decreased (↓) and increased (↑) concentrations. The control positive treated with ciprofloxacin showed 4.25 mm zone while control negative treated with DIW exhibited 0 mm. The bactericidal efficacy %age was raised from 24.7 to 43.5% and 43.5–54.1% for ZO-doped NPs at minimum and maximum concentrations, respectively (Fig. 9c). Similarly, 15.3–23.5% efficacy resulted at maximum concentration only for AS-doped NPs (Fig. 9d). In summary, CuO doped with ZO extract and optimized at 6 mL:1 manifested higher bactericidal potential against pathogenic *E. coli* of bovine mastitis origin (*p* < 0.05) as shown in Fig. 9a, b.

**Table 1:** Bactericidal activity

Etiology		500 μg/50 μl					1000 μg/50 μl				
	Material	1	2	3	4	5	1	2	3	4	5
Pathogenic *E. coli*	ZO:CuO	1.05	1.85	1.65	4.25	0	1.85	2.3	2.2	4.25	0
	AS:CuO	0	0	0	4.25	0	0.65	1	0.95	4.25	0

^1^(3 mL:1), ^2^(6 mL:1), ^3^(12 mL:1), ^4^Ciprofloxacin, ^5^DIW

**Fig. 10:**
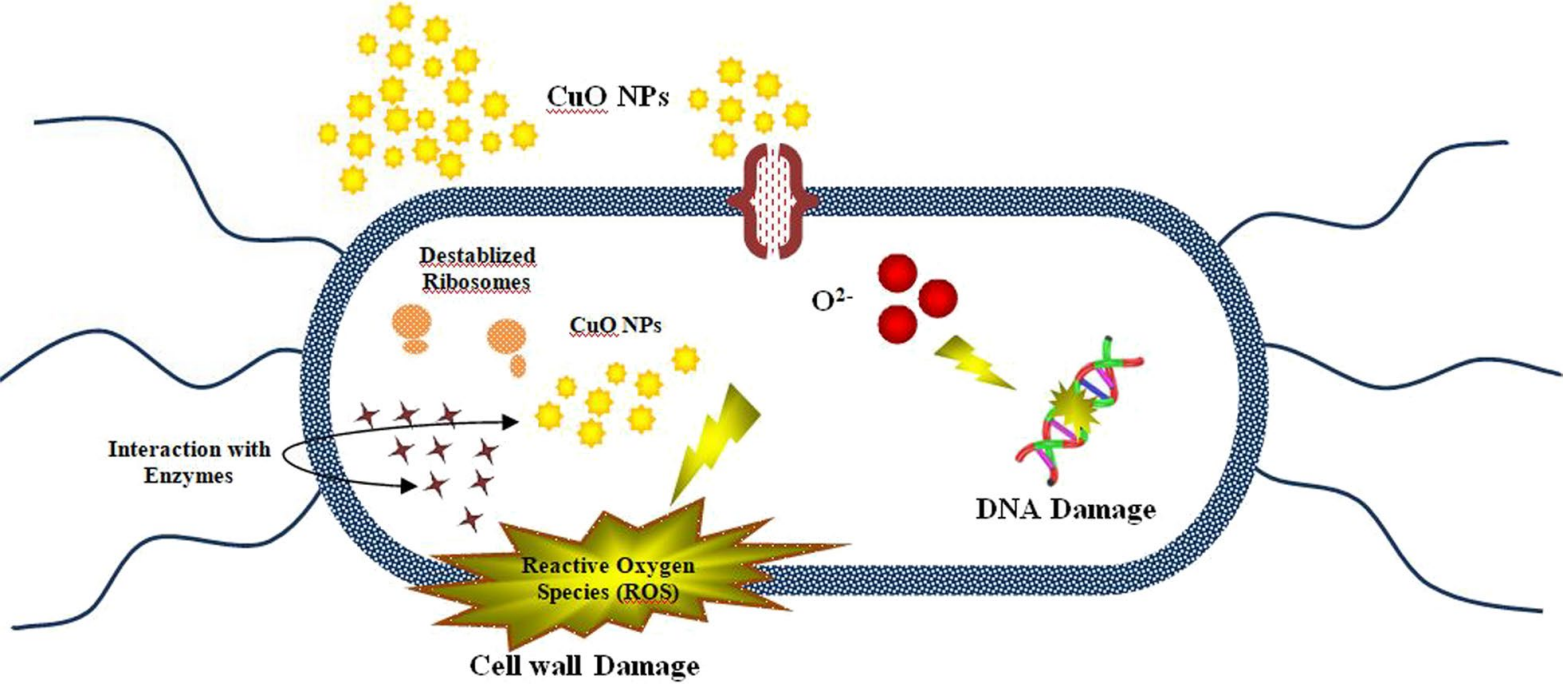
Illustration of bactericidal action of CuO NPs

Bactericidal potential of nanoparticles is dependent on the size of NPs, morphological structure and surface-to-mass ratio. Reactive oxygen species (ROS) are thought to be responsible for the formation of zones of inhibition by CuO nanoparticles [58, 59]. Denaturation of cell protein resulted through generation of detrimental reactive oxygen species (ROS) [60]. Some reactive species exhibited momentous roles in the photocatalysis such as hydroxyl and superoxide radicals and holes [61]. Synthesis of reactive oxygen species (ROS) and release of metal ions are the major features manifesting the structural changes of enzymes and proteins consequently resulting in irreparable damage to DNA and subsequent bacterial death [62]. Similarly, oxidative stress produced by reactive oxygen species (ROS) is considered to be the major contributor to photocatalysis [63]. ROS production is inversely proportional to the size of nanoparticles, i.e., smaller the size of NPs, higher is the ROS production which consequently damages the bacterial membrane resulting in extrusion of cytoplasmic contents and DNA degradation leading to bacterial burst as portrayed in Fig. 10. At the same time, positively charged Cu interacts electrostatically with negatively charged bacterial membrane resulting in cell disintegration and finally bacterial destruction [58, 64, 65]. Two responses have been proposed as potential for the bactericidal mechanism of nanostructures. One involves better linkage between the cations Cu^2+^ and bacterial cells, leading to the formation of negativized sections and eventual collapse. The other involves electronic excitation of the CuO valance band surface via excitation. Furthermore, the electrical O_2_ reaction produces O^2−^ radicals, leading to the formation of H_2_O_2_. The generated O^2−^ species are essential for the breakdown of lipid or protein molecules on the exterior cell membrane of bacteria [58, 66].

## Conclusions

Bactericidal potential of CuO NPs doped with *Zingiber officinale* and *Allium sativum* extracts against pathogenic *E. coli* was evaluated in this study, produced with the aim of alternate, economical and effective antimicrobials. The significant role of phytochemical ingredients of ZO and AS extracts was revealed in the biogenic synthesis of CuO NPs while synergistic effects of flavonoids with CuO were found to be concentration-dependent exploiting the bactericidal potential against pathogenic *E. coli*. FTIR was performed for confirmation of ZO and AS extracts doping and XRD peaks confirmed the monoclinic phase and spherical structure with mean sizes 24.7 nm (ZO-doped) and 47.6 nm (AS-doped). Spherical morphology was confirmed with FESEM images along with an exorbitant conglomeration of CuO NPs. Embellished nanoparticles revealed higher agglomeration in TEM images with size of less than 50 nm. For doped samples with root extracts, interlayer spacing of CuO nanoparticles measured as 0.23 nm was found compatible with XRD patterns. The results of this study suggest that antibacterial potential of green synthesized CuO NPs may be anticipated as alternate bactericidal agents to redress the concerns related to antibiotics resistance and residues. It may be concluded that CuO NPs doped with indigenous herbs are economical, effective and nature-friendly antibacterial agents.

## Supplementary Information

**Additional file 1: Fig. S1**. (a–d) In-vitro antibacterial activity of doped CuO NPs (a, b) Gi doped CuO at ↓ and ↑ dose, (c, d) Ga doped CuO at ↓ and ↑ doses, respectively

## Data Availability

All data are fully available without restriction.

## Abbreviations

EDS: Energy-dispersive X-ray spectroscopy
fcc: Face-centered cubic
FTIR: Fourier transform infrared spectroscopy
G + ve: Gram positive
G −ve: Gram negative
JCPDS: Joint committee on powder diffraction standards
CuO: Copper oxide
nm: Nanometer
