# Supplementary material for: Biogenic Synthesis, Characterization and Antibacterial Potential Evaluation of Copper Oxide Nanoparticles Against Escherichia coli
Source: Nanoscale Res Lett. 2021 Sep 20;16:148. doi: 10.1186/s11671-021-03605-z (PMC8452814; doi:10.1186/s11671-021-03605-z)
Supplement: Supplementary file 1 — Additional file 1: Fig. S1. (a–d) In-vitro antibacterial activity of doped CuO NPs (a, b) Gi doped CuO at ↓ and ↑ dose, (c, d) Ga doped CuO at ↓ and ↑ doses, respectively [file 11671_2021_3605_MOESM1_ESM.docx]

**Biogenic synthesis, characterization and antibacterial potential evaluation of copper oxide nanoparticles against *Escherichia coli***

Mohsin Ali^a^, Muhammad Ijaz^a*^, Muhammad Ikram^b*^, Anwar Ul-Hamid^c^, Muhammad Avais^a^, Aftab Ahmad Anjum^a^

^a^Department of Veterinary Medicine, University of Veterinary and Animal Sciences, Lahore, Punjab 54000, Pakistan.

^b^Solar Cell Applications Research Lab, Department of Physics, Government College University Lahore, 54000, Punjab, Paksitan.

^c^Core Research Facilities, King Fahd University of Petroleum & Minerals, Dhahran 31261, Saudi Arabia.

*Corresponding Authors Email: *^b^[dr.muhammadikram@gcu.edu.pk](mailto:dr.muhammadikram@gcu.edu.pk), *^a^mijaz@uvas.edu.pk


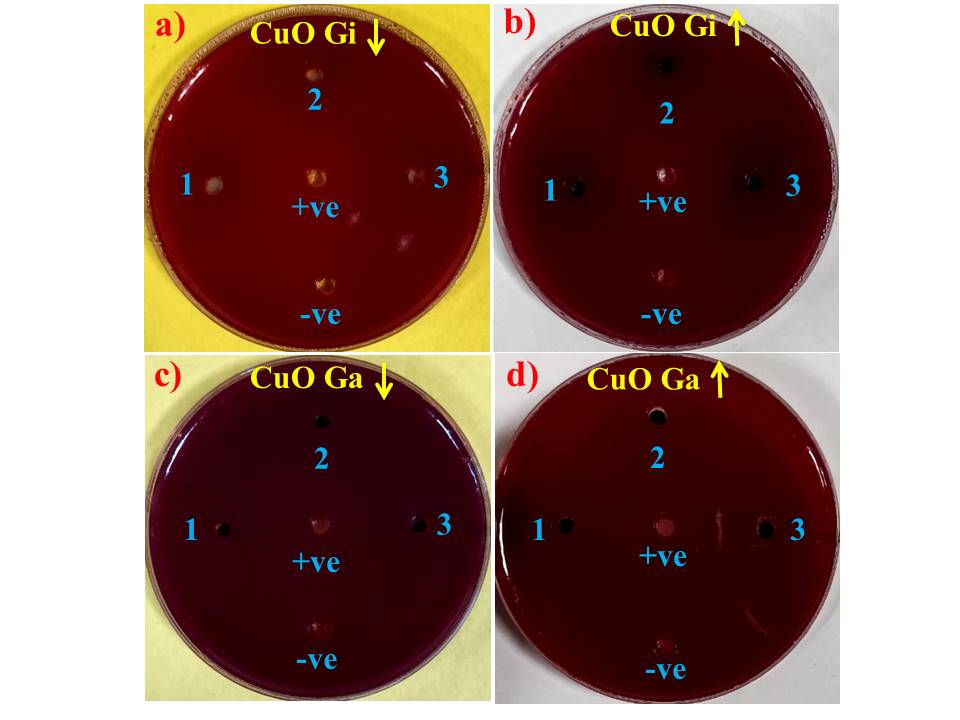


**Fig. S1:** (a-d) *In-vitro* antibacterial activity of doped CuO-NPs (a, b) Gi doped CuO at ↓ and ↑dose, (c, d) Ga doped CuO at ↓ and ↑doses, respectively
